# Secure land tenure as prerequisite towards sustainable living: a case study of native communities in Mantob village, Sabah, Malaysia

**DOI:** 10.1186/s40064-015-1329-4

**Published:** 2015-09-24

**Authors:** Gaim James Lunkapis

**Affiliations:** Faculty of Humanities, Arts and Heritage, Universiti Malaysia Sabah, Kota Kinabalu, Malaysia

**Keywords:** Sustainable, Highland, Malaysia, Native, Policy, Settlement, Sabah, Traditional

## Abstract

Sustainable livelihoods, once enjoyed by native communities, are often threatened and in danger of extinction when new regulations and other forms of restrictions are introduced. These restrictions are often promoted with intended purposes, such as protecting the environment or securing resources from encroachment. However, these acts are slowly replacing the traditional *adat* (customs and traditions), which are used to define the rights attached to the use of communal and ancestral land. This is especially true when comes to access to forest products and land, in which native communities have used for generations. What the natives see as legitimate and traditional use, the state sees as an encroachment of property; and it has now become illegal to utilise these resources. This paper presents how native communities have adapted to such restrictions and continued to live in a sustainable manner through an adaptive strategy that is in line with state policy changes. A combination of quantitative and qualitative method is used to understand the dynamics of the strategy used by the native communities to adapt to these policy changes. The findings reveal how the natives have employed an adaptive strategy in response to state policy changes. The lessons learned from this study can provide useful pointers as to how state policies, in relation to highland settlements in the state of Sabah, Malaysia, can be improved.

## Background

**Fig. 1:**
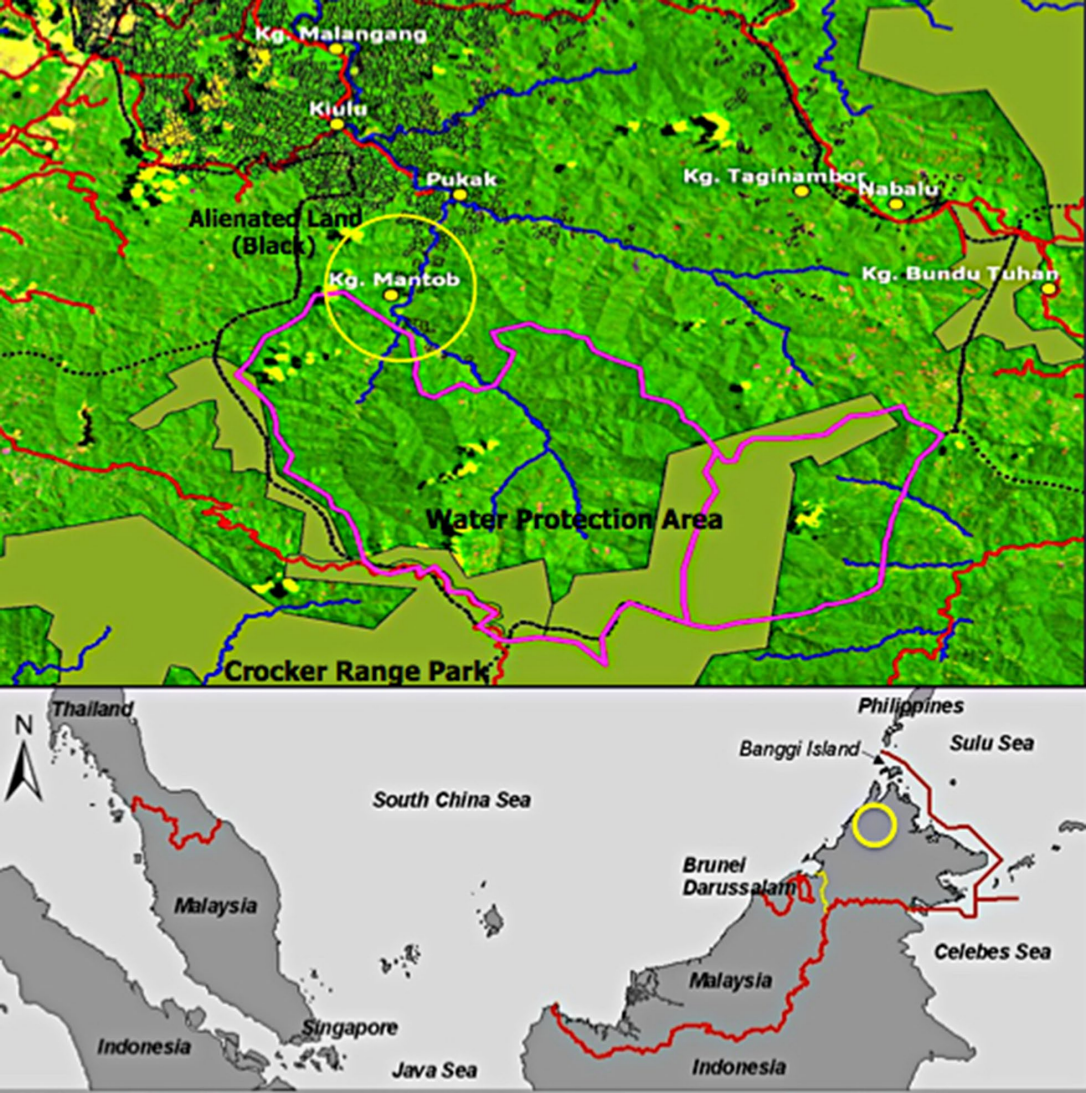
Mantob Village, the water protection zone and the Crocker Range Park

Sabah is located on the Northern Tip of Borneo, the third largest island in the world and host of the first popular television series, the Survivor (Fig. 1). Borneo’s people are a mosaic of culturally distinct native groups scattered across the highland and known for their strong interdependence with natural resources. These native peoples gained access to natural resources through kinship, local customs and through international conventions (Doolittle 2005), but different interpretations of the ways in which this access is gained may give rise to complications. There will definitely be some sort of ambiguity with regard to the historical and contemporary representation of identity between the state and the self-representation of authority and governance, and between the claims of native settlers and the state to property and resources (see for example Sanders 2006). Because of this ambiguity, and the fact that the jurisdiction of formal and informal power structures often overlap, there are often multiple social and legal institutions that can be called upon to legitimise ‘Native, State and Settler’ claims to land and resources (Hodgson and Schroeder 2002). In the case of Sabah, Malaysia, land use zoning and planning regulations and policies based on the *Town and Country Planning Ordinance*, *1950* and the *Lands and Surveys Ordinance*, *1930* have been promoted to guide future development strategies (Government of Sabah 2002). Unfortunately, the resulting changes to the access, use and ownership of land have often produced tensions and even conflicts in state-people and people-state relationships (Lunkapis 2013). It is essential that these issues be addressed and understood as a way forward towards a just and civil society.

This paper explores the sustainability aspect and adaptive strategies of Native communities in the highland settlements of Sabah, Malaysia. The historical governance of the native people of Sabah has evolved through at least several different periods (Singh 2000). Each of the periods demonstrated different restructuring scenarios of native administration, which included new laws and regulations limiting the native peoples’ access to land and natural resources. Prior to 1881, communities were confined to village-level administrations. A village might have included one or more households and was not necessarily based on kinship groupings, although this was so in some areas, especially in the remote areas of Sabah.

The administration systems of the native people were governed by the *adat* institution, which was normally headed by the *Ketua Kampung* (village head) and, in some instances, the *bobolian* (religious priest). These institutions played an important role in the decision making and other administrative matters of the native people (Appell 1968; Phelan 2003; Woolley 1953). After 1881, the various administrative systems emphasised the need for regulated control over access and land use, and thus, different sets of regulations were introduced. Most importantly, all these laws and regulations were based on economic interests, and were particularly aimed at protecting the investment potential of supplies of raw materials and plantation products to Western countries (Doolittle 2003). Despite these changes, it is argued here that the adaptive strategies employed by the natives and the fact that the state also benefited from these strategies provide a strong foundation for those native communities with potential to go for a partnership beyond the status quo of tacit hostility between customary and postcolonial governance mechanisms, and to have the capacity to shape the future of both the state and community towards just, sustainable and equitable development. The native peoples’ contribution to nation-building and statehood through their adaptive strategies has enhanced the future prospects of Malaysia to achieve its vision to become a developed nation, as demonstrated in the case study of Mantob Village.

A mainly qualitative method was used in this research, relying both on a critical review of recent working experiences (Dowling 2005; McGregor et al. 2007) and fieldwork activities focused on a case study at Mantob, a Kadazan-Dusun community in the highlands of Sabah. A combination of primary and secondary data was used for analysis, while the construction and formulation of information were influenced by the specific themes chosen for this study on sustainable highland settlements. The knowledge gained through literature reviews provided a background understanding about land use policies and current issues concerning the use of resources and access to land.

## Case descriptions

The alienation, occupation, acquisition and utilization of land in the rural areas of Sabah are regulated by the Department of Lands and Survey through the powers vested to it by the *Land Ordinance*, *1930; Land Acquisition Ordinance*, *1950*, *and the Country Land Utilization Ordinance*, *1962*. The planning for urban and rural areas is under the jurisdiction of the Department of Town and Regional Planning by virtue of the *Town and Country Planning Ordinance*, *1950*. Apart from the above, the state, from time to time, introduces new laws and policies aimed at providing greater control over the use of specific resources and access to land within the jurisdiction of specific state agencies.

As previously mentioned, the emergence of new resource policies and access controls has, to some degree, upset the native peoples’ access to their customary and everyday use of land and natural resources. In 2000, for example, the Sabah Ministry of Agriculture Development and Food Industry introduced new restrictions on the use of land above a gradient of twenty-five degrees, ostensibly to reduce upland soil erosion. Section 6 of this policy (Government of Sabah 2000: 7) states that “*Recognizing the importance of soil as a resource vital for agriculture, efforts will also be undertaken to maintain the soil eco*-*system of the state to ensure its proper utilization and conservation. As such, steep terrain of between 20 and 25 degrees slope, as defined in the* “*Soils of Sabah (1975) Study*”, *will only be allowed for development under environmentally*-*friendly methods*”. The downstream impact of this policy was that several other government departments adapted this section as their own, including the Department of Town and Regional Planning, the Department of Forestry, the Department of Irrigation and Drainage, and the Department of Environmental Protection. In fact, the ‘25° slope rule’ has now been adopted by the state as one of its key considerations when dealing with land-related development and approval of land alienation. Sixty-four percent of the total land mass of Sabah has a gradient of above twenty-five degrees (Thomas 1976), as shown in Table 1.

**Table 1:** Slopes and land area of Sabah

Slope of land	Percentage (%) of land area
0^o^ (tidal swamp)	6
0^o^ (freshwater swamp)	5
0°–5°	6
5°–15°	6
15°–25°	13
Above 25°	64
Total	100

Thomas (1976), ‘the land capability classification of Sabah’

Historical records show that quite a large number of native people live in highland areas with slopes of more than 25 degrees but possess no land title, either as individuals or as communities. Highland settlements are quite common in Sabah and have been noticeable ever since the colonial period, as recorded by the Cambridge North Borneo Expedition in 1956. Wood and Moser (1958:63) stated that “*In this area, the altitude is not less than 2500 feet and most villages lie over 3000 feet. The tropical climate is modified by these heights and there is a more noticeable diurnal range than in Tambunan*, *with colder night temperatures*”. The area described by Wood and Moser lies along the Crocker Range between Tambunan, Tuaran and Ranau District (Wood and Moser 1958). Some of the villages identified in the report and still existing today are Kirokot, Tenompok, Pinsoririan, Purakogis, Liwantai, Tiong and Randagong.

Arising from the 25° slope policy, the scenario on the ground concerning access and use of land has changed and disrupts the pre-existing *adat* systems. All previous activities and normal daily routines have now become unlawful. The only means for the natives to secure their legal rights to continue earning their livelihood in the highland areas is to go through a highly regulated land application process. Although the native peoples have accepted the new rules of the Land Ordinance and have officially applied for titles to all land owned under the *adat* systems and customary tenure (Long et al. 2003), they still face difficulties in having their previous ownership of land officially recognized. This policy has also defeated the provision under Section 15 of the *Sabah Land Ordinance, 1930*, which clearly identifies categories of lands that can be alienated to native people, especially *ladangs* and forest gardens.

In Sabah, *ladangs* (forest gardens and land used for swidden agriculture) are quite often misunderstood in terms of both their uses and concepts. *Ladangs* are normally used as sources of timber and non-timber forest products, and are particularly important to the native peoples as they are able to generate a cash income and to function as a ‘safety net’ in times of hardship, thus contributing to the improvement of rural livelihoods (Brodbeck et al. 2003). Parts of the *ladangs* are used for subsistence farming and are often left fallow for several years at a time for ecological reasons, in accordance with customary practices and traditional ecological knowledge. Because of their significance, *ladangs* became the property of and source of livelihood for the native peoples. The Cambridge Expedition (Wood and Moser 1958: 63) in 1956 to various parts of the highland settlements of Sabah (then called North Borneo) recalled “*Each family owns at least one ladang and rich ones may own as many as eight. Land is always inherited within the family…since some ladangs lie fallow for 15*–*20* *years and, although the owning family may no longer live permanently in the village, the land they once cultivated and the trees on it still belong to them*”.

Majid Cooke (2006) argued that the policies introduced and adapted by the state may have targeted land owned under Native Customary Rights, as seen in a number of land-related policies. Native communities, on the other hand, were unaware of state edicts since most did not have access to government gazettes (Majid Cooke 2006). Firstly, the 25-degree slope has become the reference point for the District Land Utilization Committee (LUC), a one-stop committee to process all land applications in a given district. Previously, if land fell within the greater than 25-degree gradient category the native’s land application was immediately rejected regardless of *adat* ownership and local histories (Government of Sabah 2001), thus effectively erasing the native’s customary rights, and at the same time reverting the land to state ownership. This has been improved in many ways under the PANTAS program (Sabah Times 2013), and customary lands are now considered for land applications. Secondly, as a result, native peoples find themselves unable to acquire financial grants or loans, either from the state or from commercial finance institutions, because they do not have proof of land ownership, in turn preventing native peoples from moving out of poverty (Ye 2006). Thirdly, the affected highland areas have been targeted for conservation policies, and most likely will be set aside as protected areas (Daily Express 2005), resulting in further restricted access to and use of land and forest products.

As a result of this pattern of circumstances, a significant number of land-related issues in Sabah were recorded in the SUHAKAM (Human Rights Commission of Malaysia) Annual Report 2005 (SUHAKAM 2006). According to this report, a total of 781 complaints were received in 2005, of which 371 or 48.1 percent were on land matters (see Table 2). Most of these were related to native customary rights or native land applications. In some cases, these native customary rights issues were associated with villages located within forest reserves and state parks. Table 2 provides a detailed breakdown of the complaints received.

**Table 2:** Complaints received by SUHAKAM in 2005

No.	Nature of complaints	Number of cases
1	Land matters	376
2	Birth certificate/identity cards/passports	154
3	Discrimination in the workplace	58
4	Police matters	46
5	Basic amenities	37
6	Employee provident fund/insurance/SOSCO/pension	27
7	Legal matters	17
8	Housing matters	13
9	Education matters	3
10	Others	50

SUHAKAM annual report 2005

Thus far, SUHAKAM has submitted several comprehensive reports containing recommendations with regard to the native peoples to the Malaysian Government. These recommendations cover forest and land rights, rights to resources, rights to development, and rights to self-worth and dignity (Yaang 2006). Unfortunately, the responsible agency has not acted on any of these recommendations, and this has given rise to further concerns about the sincerity of the current administration in tackling the problems faced by the native peoples in Sabah.

Recent developments and initiatives indicate that the State government, through the Land and Survey Department, has dealt with 615 registered cases of land claims related to Native Customary Rights (NCR) involving 1164 claimants from 1997 until February 2013. Of the total, 365 were confirmed cases, 70 cases were rejected as being without basis, while 180 cases are still being acted upon by the Sabah Native Land Administration Mobile Unit or PANTAS, which was set up in 2012 (New Sabah Times 2013).

### Case study: Mantob village

The village of Mantob, located in the interior of Kiulu, is under the administration of the Tuaran District Office, Sabah. This village was established in the 1960s by the migration of people from Tolungan, Gonipis and Nuluhon, about 50 km upland inside the Crocker Range Park (see Fig. 1). Their reason for moving to Mantob then was to seek a better livelihood by settling in an area closer to the market and with access to more land and community facilities, especially health services and schools. Mantob is located on a hillslope overlooking the Tuaran River, which lies in a narrow valley used mostly for planting wet paddy.

The surrounding area is rather hilly, with scattered cultivated forest and rubber gardens. Some households have official native titles to their lands but most have applied for them. The villagers rely mainly on subsistence farming and rubber tapping, although some of them are employed by state agencies as teachers and office workers. Most, however, grow paddy combined with some cash crops such as vegetables, banana and ginger. The villagers rarely practise shifting cultivation as they prefer to cultivate wet paddy and rubber. During the fruit seasons, most villagers are kept busy tapping rubber, harvesting fruits from forest gardens and collecting forest products to be sold in the nearby market for cash.

Although Mantob is administered under one Village Security and Development Committee and Village Head or *Ketua Kampung*, physically the village of Mantob consists of two settlement areas about one kilometre apart, separated by a small hill. It is inhabited mostly by Kadazan-Dusun people. According to the Tuaran District Office, the number of inhabitants totalled 280 in 2004 with about 32 households. The average size per household was 7 persons. The village is made up of two village reserves; one at 26.2 acres, where most of the villagers live, and the second, about 6 km north, of about 50 acres.

The forest around the village is characterised by different types of land use and forest coverage. Near the village, most of the land has been cultivated with forest gardens and turned into anthropogenic forests, which become a source of income during the fruit season. Further north of the village is the water protection zone established under the newly completed Tuaran District Land Use Plan. The communities from Mantob Village used to enter this area to collect mainly forest products, especially rattan and bamboo, which are essential raw materials for domestic handicraft.

## Discussion and evaluation

### Land tenure

Based on the data collected, 24.81 % of the total land owned by the communities is titled land while 73.64 % is still under land application (Table 3a). With respect to the number of pieces of land owned, the data indicate (Table 3) that each family member owns more than one piece. Most of them own between 4 (30.23 %) and 5 (52.71 %) pieces of land, while some own more than six parcels (6.20 %). The size of each plot ranges from 5 to 21 acres each with an average of 15 acres per plot, but none of the lots are larger than 21 acres. Most of the titled land is between 11 and 15 acres each (78 %), and the parcels of land under land application are also generally 11–15 acres each (81.23 %), (see Table 3).

**Table 3:** Land ownership in Mantob village

Land ownership	Number (acres)	Percent
a		
Titled land	32	24.81
Land application (LA-pending)	95	73.64
Customary owned (not applied)	2	1.55
Total	129	100.00

Number of pieces per person	Number (person)	Percent
b		
1	0	0.00
2	3	2.33
3	11	8.53
4	39	30.23
5	68	52.71
6 or more	8	6.20
Total	129	100

Size of titled land (acres)	Number (person)	Percent
c		
0–5	0	0.00
6–10	13	22.03
11–15	46	77.97
16–20	0	0.00
21 and above	0	0.00
Total	59	100

Size of land under application (LA)	Number (person)	Percent
d		
0–5	0	0.00
6–10	10	15.63
11–15	52	81.23
16–20	2	3.13
21 and above	0	0.00
Total	64	100

Land suspected inside WPZ^a^	Number (pieces)	Percent
e		
0–5	0	0
6–10	2	5.88
11–15	30	88.24
21 and above	2	5.88
Total	34	100

Fieldwork data collection

^a^Exact demarcation of water protection zone (WPZ) is not known

### Land use

The land in Mantob Village is used predominantly for agriculture, and this includes rubber gardens, fruit orchards, paddy fields and mixed gardens. About 14 % of the rubber gardens are less than 5 acres, 26 % are between 6 and 10 acres, while the majority are between 11 and 15 acres. None of the rubber gardens are larger than 20 acres (Table 4). Almost all the fruit orchards are less than 5 acres (89.80 %), but field interviews revealed that some families owned more than one plot scattered in different places, and these were chosen because of the quality of the soil and the suitability of the location (Table 4b). The average size of the mixed gardens is also about five acres each (97.73 %, see Table 4c).

**Table 4:** Land use in Mantob village

Rubber gardens (acres)	Number (person)	Percent
a		
0–5	6	13.95
6–10	11	25.58
11–15	26	60.47
16–20	0	0.00
21 and above	0	0.00
Total	43	100.00

Fruit orchards (Anthropogenic forest)	Number (person)	Percent
b		
0–5	44	89.80
6–10	5	10.20
11–15	0	0.00
16–20	0	0.00
21 and above	0	0.00
Total	49	100

Mixed Gardens (ginger, banana, pineapple, etc.)	Number (person)	Percent
c		
0–5	43	97.73
6–10	1	2.27
11–15	0	0.00
16–20	0	0.00
21 and above	0	0.00
Total	44	100

Active land (currently planted and in use)	Number (person)	Percent
d		
0–5	30	37.50
6–10	35	43.75
11–15	15	18.75
16–20	0	0.00
21 and above	0	0.00
Total	80	100

Fieldwork data collection

The mixed garden products include vegetables, pineapples, and ginger, which are used mostly for the daily consumption of the family, although surpluses, especially of ginger and pineapples, are sold in the nearby local market. Most of the weeding and planting in the mixed gardens are done by the women, but the harvesting and carrying of the garden products are shared equally between the men and women. Because of the variations in land use, the land owned by family members is very active throughout the year. About 37.5 % (Table 4d) of the active lands are less than 5 acres, while 43.75 % of them are between 6 to 10 acres, and 18.75 % are between 11 and 15 acres (Table 4d). As for wet paddy planting, most families plant only once a year as they need to allocate their time for other activities that are capable of generating a cash income, especially rubber tapping and the collection of fruit from orchard gardens.

Mantob Village has several different types of managed land units: *tumoon* are cultivated plots containing staples such as rice; *tomulok* is fallow land, although resources continue to be extracted for a few years, especially banana, pineapple and cassava; a *kebun* is a fruit garden; *gouton* is a plot of land where resources such as rattan, wood products and medicinal herbs are harvested. *Puru* is an area of secondary or primary forest that is located further, around four kilometres or more, from the village area. *Puru* is mainly used by the villagers for forest and non-forest products, such as for collecting wood and rattan and also for hunting wild animals. Most of these areas now fall under the Crocker Range Protection Zone. The *rizab kampong* is the community-owned reserve land that accommodates residential areas and grazing land for livestock, especially buffaloes. The rivers provide fish, while the vegetation growing in this area is used for building materials, especially *rumbia* and *polod*, which are used as roofing materials for local huts and to make handicrafts.

### Traditional land use and conservation zone

One of the great concerns expressed by local communities during fieldwork activities was with regard to the decision of the state to create another layer of conservation, the Water Protection Zone (see Fig. 1), which was introduced in 2004 under the Tuaran District Land Use Plan, and the slope policy introduced in 2000 under the Second Sabah Agricultural Policy (Government of Sabah 2000). Local communities feel that this might delimit their traditional use of land as a major portion of their ancestral land had already been acquired for the Crocker Range Park in 1985.

The exact boundary of the Water Protection Zone has not been surveyed but local residents are already experiencing the pressure, as their land applications have not been processed. Based on this, respondents were asked to identify if their land falls within the proposed zone or not. The result revealed that about 32 pieces of land, ranging from 6 to 20 acres each, are suspected to fall within the Water Protection Zone (Table 3). Some have been issued land titles but some still remain under the status of land applications. It is not known when these land applications will be processed and the titles given.

Apart from issues concerning the Water Protection Zone, the 25°-slope policy is also a great cause for concern as Mantob Village is located in the highlands region. As previously stated, this policy prohibits any activities in the highlands area, which is currently being enjoyed as the main source of livelihood for the natives of Mantob Village. A brief look at the map from the Department of Survey and Mapping revealed that Mantob Village is within the 1500–3000 m contour line. It was also observed that due to the sharp land gradient no road had been constructed to directly link individual family dwellings, except for footpaths. There is, however, a gravel road through the southern part of this village, which has only been accessible since 1986.

### Rubber gardens

Although the market price for rubber fluctuates, rubber tapping has always been a favourable source of cash income. It was noted during fieldwork that the Sabah Rubber Industrial Board (SRIB) collects rubber sheets from Mantob and the nearby villages once a month. At the time of this research in 2010, each family was earning an average of RM 1800 (1 US$ = RM 3.60) in any given month. In response to the efforts of the state to promote ecotourism as an income diversification strategy, Mantob Village started a homestay programme in 2000. One of the activities highlighted by the programme is rubber tapping and rubber planting. In line with the SRIB strategy to promote the smallholders scheme, several families in Mantob Village have identified several plots of their *ladang* for the planting of rubber. Hybrid seedlings and fertilizers are supplied by SRIB at a subsidized rate.

Through observation, this case study revealed that crop diversification is an important strategy used by local communities to participate in the market economy, and it is capable of providing good economic returns. Local communities have adapted current state policies to their own advantage and have learned to accept such policies into their daily livelihood. Unlike other parts of Sabah, where the younger generation has left in search of job opportunities elsewhere, Mantob Village seems to have a fair representation of all ages. It was also observed that shifting cultivation is no longer practised as each family has opened up a sizeable permanent plot for wet paddy cultivation.

### Land applications

The fieldwork data indicated that local communities have applied for all available lands previously owned according to *adat* systems. However, until such land is surveyed and given a title deed, the land is considered as state land and anyone can apply for land alienation. The village community of Mantob learned this when they received a letter from the Tuaran District Land Office stating that their land applications could not be processed because the land applied for overlaps another application. Out of frustration, Gaman (not his real name) did some investigations and found out that a private limited company, *X Company Sdn Bhd*, had submitted an application dated Jun 1998 for about 2000 hectares, which will encompass an area just outside the boundary of Mantob Village. Gaman’s land application falls within this area (personal communication).

Gaman and several others from Mantob wrote a letter of objection to the land office. At the time of this fieldwork, this author was informed that the *Ketua Kampong* (village head) of Mantob had been given a verbal assurance by the land office that the land application by *X Company Sdn Bhd* would be rejected. However, Gaman received another letter from the land office stating that the land he applied for is too steep and is unsuitable for agriculture, and as such his application would not be processed. Field investigations revealed that Gaman had applied for the land in May 1982.

Subsequently, Gaman wrote to the land office stating that the land he had applied for was actually inherited from his parents, who had planted the land with rubber and fruit orchards. They survived and earned a living from this land and had grown vegetables and other cash crops. The field investigation revealed that the rubber grown in Gaman’s land was about 15 years old, but his family had used the land *(ladang)* before for short-term crops and vegetable gardens. Currently, Gaman’s land provides his family with a good cash income through rubber tapping and the cultivation of other garden products. When asked how often Gaman visited the land office, he said “*Whenever I visit the government offices, they tell me that the forest belongs to the government, and that I have no rights to the land of my ancestors. They say that if I want the land, I just have to apply for titles, but I have already done that years ago. Yet I am still waiting. I am too old no*w” (Personal communication with Gaman in 2010).

It is understood that the size of the land in Gaman’s land application is 15 acres but the actual area of his plot is about 25 acres. Some areas are quite steep and fall within the river reserve. Recent communications with Gaman in 2013 revealed that he had received a letter from the land office indicating that his land was approved for surveying. At the time of this fieldwork (2010), Gaman was 62 years old. It is important to mention here that the administrative process for approving land applications often takes a long time (Cleary 2002; Doolittle 2005). Finally, it was observed that claims for large portions of the customary lands have been applied for by family members but they all tell stories that are similar to Gaman’s experiences.

## Conclusion

The experiences of the natives in Mantob Village are representative of the daily experience of communities living in the highlands of Sabah. The secure ownership of land derived from the Land Ordinance is slowly replacing the traditional *adat,* which used to define usage rights and land ownership. Traditional access to land allowed native peoples to grow their own food crops, in addition to gardening and the gathering of forest and non-forest products to make ends meet.

The adoption of cash cropping, easily available through free access to and use of land, was able to meet the native peoples’ cash needs. Hence, the land provides the critical resources needed for the economic and financial security of the local communities. However, the land can only exist in a finite quantity, and most of the communities in the highland settlements have officially applied for titles to all the available land in the area, and land use zones such as the Crocker Range Park, the Water Protection Zone and other such zones are constraining and reducing the space available for cultivation and land ownership to provide for local livelihoods (see Fig. 1).

The fact that several land-related ordinances coexist with other laws and regulations, including the traditional *adat* (custom or customary law) systems of local communities, means that there are multiple interpretations in terms of access to and use of land. The agenda of the state and that of the people on land use has been conflicting and contradictory. Historically, Sabah was ruled by the North Borneo Chartered Company (NBCC) from 1881 to 1946. From 1946 until Independence in 1963, North Borneo was a Crown Colony of England (Singh 2000). Both the state and the natives have taken advantage of the different sets of laws in advancing their respective claims to access rights and land ownership (Doolittle 2005), as was also demonstrated in this paper. This, in many ways, has revealed the sustainability of the native communities living in the highland areas of Sabah.

As for the theoretical aspects, the individuals or groups that ‘hold power’ within planning circles can systematically exclude particular groups from the decision making process (Hillier 1993). Similarly, land use planning can be a useful tool to pursue a predefined agenda while hiding behind the true vision of planning for the benefit of the dominant group. It is understood that planning has been a central idea in the developmentalist agenda and is used as a basic strategy for achieving social, political and economic goals (Howitt 2001). Similarly, the planning and the implementation of policies concerning local communities in the highland settlements of Sabah may have been constructed and communicated through the District Land Use Plan with the aim of providing a more efficient administrative mechanism for state conservation initiatives. The views of local communities were only used to show there was ‘consultative participation’ (Pimbert and Pretty 1997) during the planning process and merely to fulfil the statutory requirements spelt out under the ordinance.

In addition to the ‘planning instruments’ and ‘local livelihood strategies’ (Howitt and Lunkapis 2010) discussed above, it has been shown in this paper that contradictory definitions and understandings of livelihood strategies have become a stumbling block in raising the standard of living of the native communities in the interior of Sabah. The two examples mentioned above are access to and use of land above the 25° gradient, and land ownership based on *adat* and on the Land Ordinance.

Although the 25° slope policy was originally conceived by the Department of Agriculture, the enforcement of this policy is through the land application approval process. While hiding behind environmental consciousness and soil fertility standards, the recommendations from the Department of Agriculture with regard to the soil conditions of the land applied for by the natives play an important role and can determine the outcome of the decision of the state to award land rights to the natives. The slope policy was singled out as one of the reasons for the many land applications that are pending, as seen in Gaman’s example. Native communities, on the other hand, have taken advantage of the policy of the Sabah Rubber Industrial Board (SRIB) to plant rubber trees in the highlands and in turn, this has benefited both the state and the people (Kaur 2006). This policy allows native communities to open up land above the 25° gradient for rubber gardens with aid from the state in the form of technical support, subsidies, seedlings and fertilizers. It is understood that land applications for rubber gardens will be pursued through the SRIB. This opportunity has seen many family members of Mantob Village opening up *ladangs,* which were traditionally owned based on *adat,* for rubber gardens.

Besides the two pressing issues mentioned above, the natives of Mantob Village have gained many opportunities through state policies. Current initiatives to promote ecotourism in the form of homestay programmes provide an excellent example. Although the real intention of the state is environmental education, the people see this programme as an opportunity for earning extra cash income while promoting their culture and way of life to the outside world. The current policies and political climate have seen the national and state governments aggressively advertising Sabah as a world destination for ecotourism. This initiative has, in a way, attracted attention to the native communities living in the highlands of Sabah, which in turn promotes the sustainable livelihood of the native communities, as shown in this case study on Mantob village.
